# A general non-rectangular hyperbola equation for photosynthetic light response curve of rice at various leaf ages

**DOI:** 10.1038/s41598-019-46248-y

**Published:** 2019-07-09

**Authors:** Junzeng Xu, Yuping Lv, Xiaoyin Liu, Qi Wei, Zhiming Qi, Shihong Yang, Linxian Liao

**Affiliations:** 1 State Key Laboratory of Hydrology-Water Resources and Hydraulic Engineering, Hohai University, Nanjing, 210098 China; 2 College of Agricultural Engineering, Hohai University, Nanjing, 210098 China; 3 School of Hydraulic, Energy and Power Engineering, Yangzhou University, Yangzhou, Jiangsu 225009 China; 4 Department of Bioresource Engineering, McGill University, Montreal, PQ H9X 3V9 Canada

**Keywords:** Light responses, C3 photosynthesis

## Abstract

Photosynthetic light response (PLR) curves of leaves are usually fitted by non-rectangular hyperbola (NRH) equation, and those fitted NRH parameters may change with leaf aging. The objectives of this study were 1) to reveal the response of NRH parameters of rice leaves, light-saturated net photosynthetic rate (*P*_nmax_), quantum yield of assimilation (*φ*), dark respiration rate (*R*_d_) and convexity of the curve (*k*), to leaf age; and 2) to improve the performance of NRH equation in simulating the PLR curves for leaves at various ages. The PLR for rice leaves at ages of 3–53 days were measured, and the general NRH equation was developed by incorporating the relationship between NRH parameters and leaf age into the NRH equation. The results showed that the NRH parameters of *P*_nmax_, *φ* and *R*_d_ increased rapidly to maximum at approximately 10 days and then declined linearly toward the age of 53 days. However, the value of *k* was not sensitive to leaf age. The general NRH equation can be used to simulate leaf PLR continuously along with leaf aging.

## Introduction

Leaf photosynthetic light response (PLR) is the fundamental for understanding photosynthetic process driven by photon energy^1–3^, and for modelling net primary productivity or net ecosystem exchange^4,5^. Numerous mathematical functions have been used to describe the PLR curves, such as Michaelis-Menten, Mitscherlich, hyperbolic tangent, rectangular hyperbola and non-rectangular hyperbola (NRH) equations^6^.

Leaf photosynthetic characteristics were influenced by various leaf traits, including leaf nitrogen content, leaf chlorophyll content, specific leaf mass and leaf position^4,7–13^. Consequently, leaf PLR curves, as well as parameters in the PLR equations, varied greatly among leaves or varieties^14–16^. Incorporating those influential factors into PLR equations was important for either understanding the plant PLR or modeling plant photosynthesis at different spatial scales^17,18^. Some of those factors have been incorporated into different PLR equations. Leaf nitrogen content and specific leaf mass for herbaceous and woody angiosperms were found highly correlated with PLR parameters, and the interspecific PLR curves were established by linking the PLR parameters of both Mitscherlich and Michaelis–Menten functions to leaf nitrogen content and specific leaf mass^3,14^, which were tested to be accurate in depicting PLR curves among species and individual plants. SPAD value (a reliable indicator of leaf chlorophyll content)^19–21^ was incorporated into the NRH equation to build a general PLR equation, which can be used to describe PLR curves of rice leaves with different SPAD values^22^. Those researches provide marvelous cases for improving the performance of PLR equation among species, varieties, plants and leaves, which is enlightening for future research.

Furthermore, leaf age was also an important factor affecting leaf photosynthetic traits, owing to changes in both leaf traits^23–25^ and biomass sink-source relation^26–28^ along with leaf aging from leaf appearance to senescence. The declining tendency in net photosynthetic rates (*P*_n_) was observed over a wide variety of species with leaf aging from full leaf expansion to senescence^11,29,30^. For rice, measured light-saturated *P*_n_ reached the maximum at fully developed stage and then declined gradually as leaves senesced^31^, or declined from the top (young leaves) to the basal (old leaves) within rice canopy^7,32^. Thus, it was well known that *P*_n_ varied among leaves at various ages, yet there was no results discussing the changes in PLR parameters during leaf development. As a result, almost all models ignored the variation of leaf age within the canopy, and treated all leaves with the same PLR parameters calibrated based on leaf scale measurement in calculating *P*_n_ at canopy scale^1^.

Insight into the effect of leaf age on photosynthetic traits will provide basic information for either modelling leaf photosynthesis continuously along with leaf aging, or upscaling leaf photosynthesis to canopy scale by considering the variation of leaf age within canopy. To reveal the impact of leaf age on rice PLR curves and their parameters, the NRH equation was established first for each specific leaf based on PLR data collected from rice leaves at various ages. Subsequently a general NRH equation, capable of simulating PLR curves for leaves at various ages, was constructed by considering the impact of leaf age.

## Results and Discussion

### Leaf SPAD values with leaf aging

**Figure 1:**
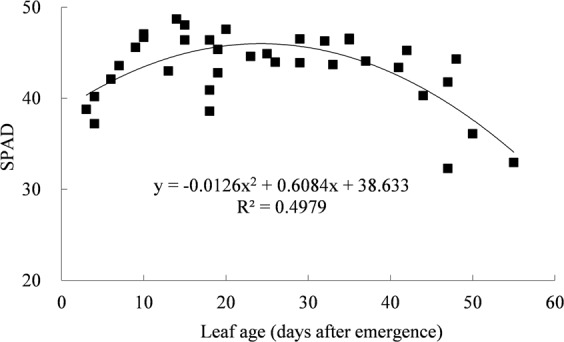
Rice leaf SPAD varied along with leaf aging.

Leaf SPAD value was considered as the indicator of leaf chlorophyll level, and highly related to leaf photosynthetic traits. Rice leaf SPAD readings varied in three stages with leaf aging, initial development, fully functional and senescence periods (Fig. 1). The initial development period lasted for approximately two weeks since leaf emergence. During this period, rice SPAD values increased rapidly. Then, SPAD readings were high and relatively constant in the full functional period for about 30 days. Finally, in leaf senescence stage, SPAD values decreased gradually. Similar results were reported on rice in East China by Yang *et al*.^20^.

### Measured photosynthetic light response for leaves at various ages

**Figure 2:**
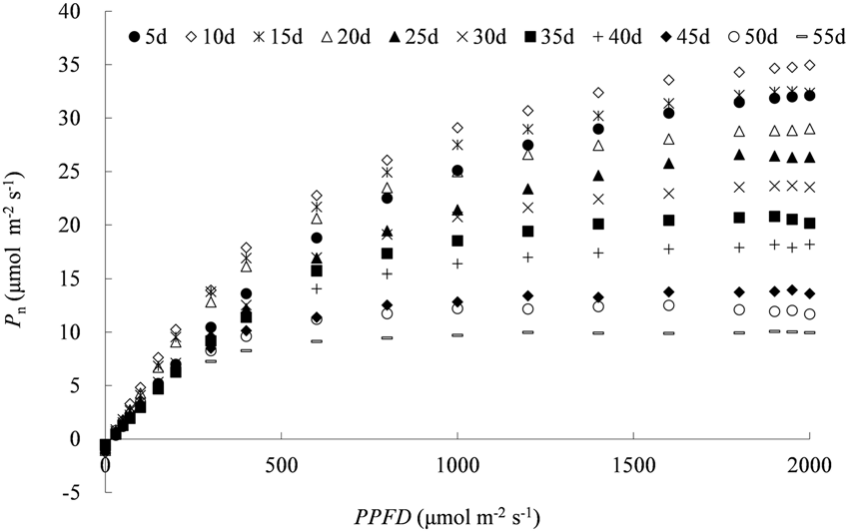
Measured response of net photosynthetic rate (*P*_n_) for rice leaves at various ages to photosynthetic photon flux density (*PPFD*) (*P*_n_ is the average photosynthetic rate for leaf age groups at *n* day, numbers “*n* d” in legend indicates the nominal leaf age for each leaf age group with ±2 days span).

These 37 independent PLR curves were categorized into 11 groups with five days as steps, by putting PLR curves with leaf age ±2 days around together. The *P*_n_ initially increased fast with the increase in photosynthetic photon flux density (*PPFD*), then slowly up to the maximum *P*_n_ (Fig. 2). Leaf PLR curves were quite different to each other among leaves at various ages. The difference in *P*_n_ among leaves was small when the *PPFD* was low, and more remarkable with increasing *PPFD*. The maximum *P*_n_ was slightly higher for the group at 10 days old (about 3~4 days after full expansion) than that at 5 days old, and then decreased gradually with increasing leaf age. The highest maximum *P*_n_ was approximately 34.89 μmol m^−2^ s^−1^ for leaf at 10 days old, it was 3.4 times that for leaf at 55 days old. The similar pattern of PLR curves was reported on cotton by Echer *et al*.^33^, who showed little effect of leaf age on *P*_n_ under low *PPFD* condition, and light-specific *P*_n_ was higher at 15-d and 30-d-old compared with 45-d and 60-d-old leaves.

### Photosynthetic light response parameters

**Figure 3:**
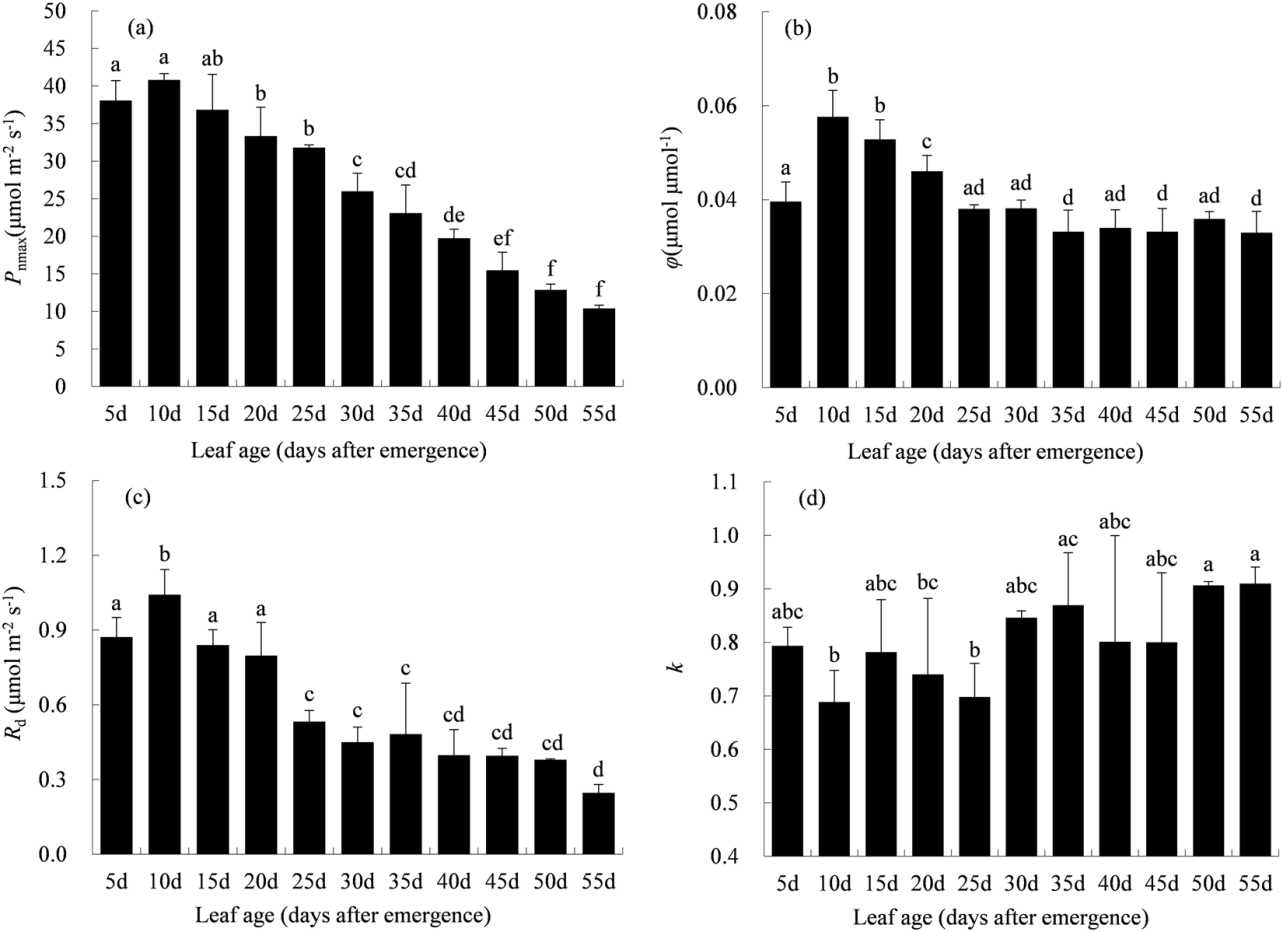
Parameters of (**a**) light-saturated net photosynthetic rate (*P*_nmax_), (**b**) quantum yield of assimilation (*φ*), (**c**) dark respiration rate (*R*_d_), and (**d**) convexity of the curve (*k*) for leaves at different age groups (different letters mean significant difference at *p* < 0.05 level with the least significant difference test).

For each of these 37 PLR curves, the NRH equation was fitted separately. The NRH equation performed well in modeling the PLR curves of individual rice leaves. The coefficient of determination (R^2^) and Nash-Sutcliffe coefficient (NS) were very high (range from 0.9892 to 0.9997 and from 0.9844 to 0.9997), and errors were very low (average absolute error (AE) and root mean square error (RMSE) range from 0.139 to 0.768 μmol m^−2^ s^−1^ and from 0.091 to 0.709 μmol m^−2^ s^−1^). Among leaf age groups, the parameters of *P*_nmax_, *φ* and *R*_d_ initially increased, reached the maximum for group at 10 days old, then decreased subsequently. The maximum of *P*_nmax_, *φ* and *R*_d_ were 40.6 μmol m^−2^ s^−1^, 0.0561 μmol μmol ^−1^ and 1.06 μmol m^−2^ s^−1^, respectively. Yet, no clear tendency was found in the parameter of *k* along with leaf age (Fig. 3).

### Correlations between parameters in non-rectangular hyperbola and leaf Age

**Figure 4:**
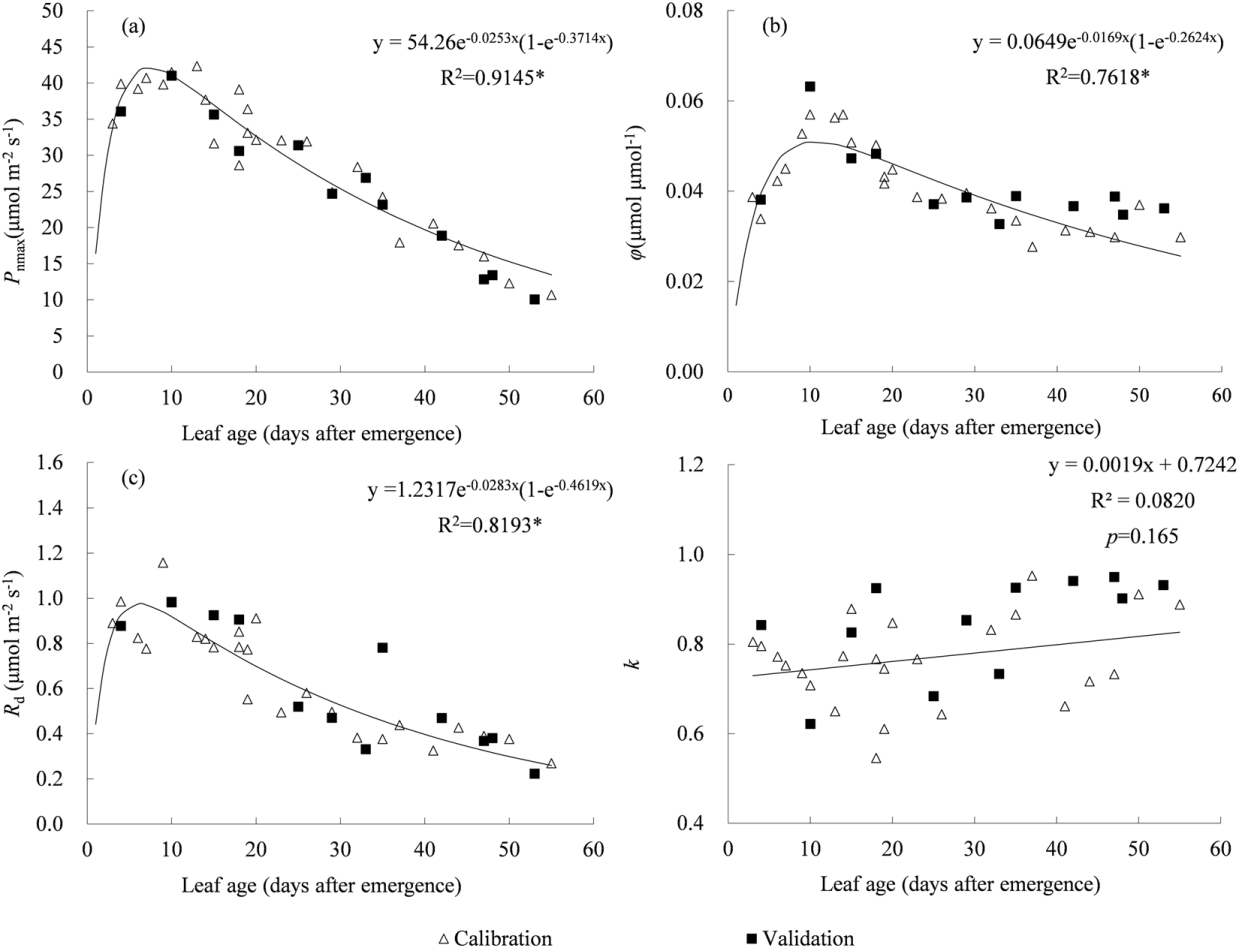
Regressions of (**a**) light-saturated net photosynthetic rate (*P*_nmax_), (**b**) quantum yield of assimilation (*φ*), (**c**) dark respiration rate (*R*_d_), and (**d**) convexity of the curve (*k*) with leaf age. (asterisk means significant relationship at *p* < 0.001 confidence level with F-test).

To describe the relationships between NRH parameters and leaf age, scatter plots and regressions between NRH parameters for each individual leaf and its leaf age, based on 25 independent PLR curves (calibration data), were shown in Fig. 4. The *P*_nmax_, *φ*, *R*_d_ and *k* were found varying in a wide range of 10.07–42.38 μmol m^−2^ s^−1^, 0.0277–0.0570 μmol μmol^−1^, 0.27–1.16 μmol m^−2^ s^−1^ and 0.5460–0.9529, respectively. The parameters of *P*_nmax_, *φ* and *R*_d_ were highly correlated with leaf age. The *P*_nmax_ varied in two distinct phases, it increased rapidly to a maximum around 10 days after leaf emergence, and then declined linearly to about 10 μmol m^−2^ s^−1^ at 53 days old (Fig. 4a). The *φ* and *R*_d_ varied in the same pattern with *P*_nmax_ (Fig. 4b,c). The parameters of *P*_nmax_, *φ* and *R*_d_ could be fitted using a positive skew equation with respect to leaf age. For parameter of *k*, it increased linearly with leaf age (Fig. 4d), but the linear relationship was insignificant (*p* = 0.165). Intuitively, the validation data (the other 12 independent PLR data) matched these curves very well (Fig. 4).

Similarly, the *P*_nmax_ for cotton got the maximum at 10–15 days after leaf unfolding and then declined linearly with leaf aging^34^. Stirling *et al*.^35^ also reported *P*_nmax_ for maize varied in a similar pattern along with thermal time (effective temperature accumulated along with time). Meanwhile, the results were quite different for parameters of *φ* and *k*. The *φ* varied independently to thermal time for maize^35^, yet it varied in a positive skew pattern for cotton^34^ and rice (in the current result). For parameter of *k*, it was found varied independently to leaf age of rice, yet it was reported varied downward parabolically for maize^35^.

Generally, leaf photosynthesis was highly related to leaf chlorophyll contents^22^. For rice, leaf SPAD increased rapidly to the maximum since leaf emergence, and kept constant at the high level for a long time during 10–40 days after leaf emergence (Fig. 1). While the variation of *P*_nmax_ did not match leaf SPAD very well, the *P*_nmax_ got the maximum and then declined although the leaf SPAD was still high (Fig. 4a). It implied that leaf traits other than chlorophyll content, including leaf structural features (thickness, mesophyll cell) and functional traits (transpiration rate, mesophyll conductance, stomatal conductance), were also highly correlated with crop photosynthetic capacity^36,37^, and changed with leaf age^28,38^. During leaf expansion period, the pigment contents increased, photosynthetic enzymes were formed, and their activities increased sharply together with the efficiencies of radiant energy utilization, electron transport chain and photophosphorylation. As a result, the *P*_nmax_ increased. But in senescing period, *P*_nmax_ decreased due to the decrease in stomatal conductance, chlorophyll content, enzyme activities, etc^39^. It indicated that leaf age might be more important than leaf chlorophyll level in determining temporal variation of leaf photosynthetic capacity.

### General NRH model considering effect of leaf age

As illustrated in Fig. 4, *P*_nmax_, *φ* and *R*_d_ varied following the positive skew patterns with respect to leaf age. Equations (1–3) were used to describe the trends of *P*_nmax_, *φ* and *R*_d_ for leaves at various ages. Then a general NRH model was constructed by incorporating the Eqs. (1–3) into the NRH equation (Eq. (4) in Methods).

1 $${P}_{nmax}=f(A){P}_{nopt}={e}^{-{d}_{1}A}(1-{e}^{-{d}_{2}A}){P}_{nopt}$$

2 $$\phi =g(A){\phi }_{opt}={e}^{-{d}_{3}A}(1-{e}^{-{d}_{4}A}){\phi }_{opt}$$

3 $${R}_{d}=h(A){R}_{dopt}={e}^{-{d}_{5}A}(1-{e}^{-{d}_{6}A}){R}_{dopt}$$

where *A* is leaf age; the parameters of *P*_nopt_, *φ*_opt_, and *R*_dopt_ represent the optimal *P*_nmax_, *φ*, *R*_d_; and  *d*_1_, *d*_2_, *d*_3_, *d*_4_, *d*_5_, *d*_6_ are coefficients. The parameters and coefficients were determined by nonlinear least-square fitting based on calibration data in Fig. 4a–c. The parameters of *P*_nopt_, *φ*_nopt_ and *R*_dopt_ were calibrated as 54.26 μmol m^−2^ s^−1^, 0.0649 μmol μmol^−1^ and 1.2317 μmol m^−2^ s^−1^, and the coefficients of *d*_1_, *d*_2_, *d*_3_, *d*_4_, *d*_5_, *d*_6_ were determined as 0.0253, 0.3714, 0.0169, 0.2624, 0.0283 and 0.4619, respectively. The value of *k* was averaged as 0.7685 (Fig. 4d).

**Figure 5:**
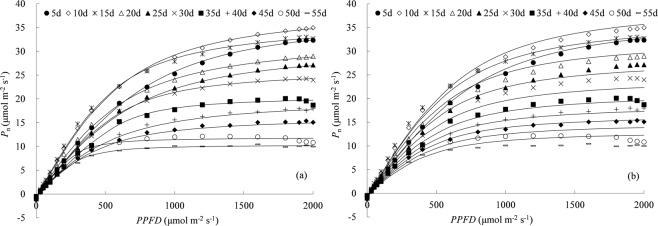
Net photosynthetic rates (*P*_n_) calculated by (**a**) the non-rectangular hyperbola (NRH) and (**b**) the general NRH equation for calibration data. Lines represent the modelled *P*_n_, symbols are the observed *P*_n_ (“*n* d” in legend is the nominal leaf age for each leaf age group with ±2 days span).

Both the NRH and general NRH equations were used to predict PLR curves over the entire range of leaf age based on each of those 25 PLR curves (calibration data) as shown in Fig. 5 and Table 1. The results indicated that the general NRH equation performed slightly inferior to the NRH equation. The average RMSE and AE were 0.902 and 0.886 μmol m^−2^ s^−1^ for *P*_n_ calculated by the general NRH equation, higher than the errors by the NRH equation (0.338 and 0.324 μmol m^−2^ s^−1^ averagely). The fitted results showed that the general NRH equation described *P*_n_ well for leaves younger than 20 days old, whereas slightly underestimated *P*_n_ for about 25–40 days old leaves and overestimated *P*_n_ for leaves older than 45 days old. Generally, good agreement was obtained between estimated and observed *P*_n_, and the general NRH equation could describe all individual PLR curves, with the R^2^ and NS ranging from 0.928 to 0.999 and from 0.873 to 0.998.

**Table 1:** Performance of the non-rectangular hyperbola (NRH) and general NRH equations in predicting photosynthetic light response (PLR).

Leaf age /d	Calibration data										Validation data				
	NRH equation					General NRH equation					General NRH equation				
	*k*	R^2^	NS	RMSE	AE	*k*	R^2^	NS	RMSE	AE	*k*	R^2^	NS	RMSE	AE
				μmol m^−2^ s^−1^					μmol m^−2^ s^−1^					μmol m^−2^ s^−1^	
3~7	0.998	0.999	0.999	0.360	0.287	1.016	0.990	0.988	1.295	1.040	0.996	1.000	1.000	0.239	0.203
8~12	0.999	0.999	0.999	0.383	0.293	1.016	0.995	0.994	0.996	0.779	1.004	0.994	0.994	1.046	0.847
13~17	0.997	0.998	0.998	0.511	0.401	0.983	0.989	0.987	1.415	1.168	1.012	0.999	0.999	0.424	0.351
18~22	0.994	0.998	0.998	0.379	0.370	1.026	0.982	0.975	1.066	1.093	0.993	0.989	0.988	1.251	1.026
23~27	0.987	0.999	0.998	0.227	0.385	0.975	0.996	0.994	0.409	0.607	1.011	0.992	0.992	0.881	0.621
28~32	0.999	0.999	0.999	0.309	0.228	0.918	0.980	0.965	1.494	1.122	1.000	0.999	0.999	0.249	0.194
33~37	0.998	0.995	0.994	0.551	0.365	0.968	0.967	0.967	1.424	1.070	0.937	0.986	0.975	1.025	0.906
38~42	0.978	0.999	0.997	0.123	0.314	0.975	0.999	0.997	0.123	0.286	0.881	0.986	0.937	1.784	1.534
43~47	0.972	0.999	0.996	0.156	0.321	1.010	0.998	0.998	0.271	0.222	1.072	0.919	0.853	1.772	1.553
48~52	0.995	0.989	0.989	0.458	0.399	1.069	0.928	0.873	0.123	1.278	1.057	0.963	0.936	0.189	1.062
53~57	0.997	0.995	0.995	0.261	0.196	1.116	0.959	0.880	1.310	1.086	1.157	0.889	0.657	2.050	1.803
Average	0.992	0.997	0.997	0.338	0.324	1.007	0.980	0.965	0.902	0.886	1.011	0.974	0.939	0.992	0.918

*k*, R^2^, NS, RMSE and AE denote slope of linear regression, coefficients of determination, Nash-Sutcliffe coefficient, root mean square error and average absolute error of net photosynthetic rates calculated based on the NRH and general NRH equations.

**Figure 6:**
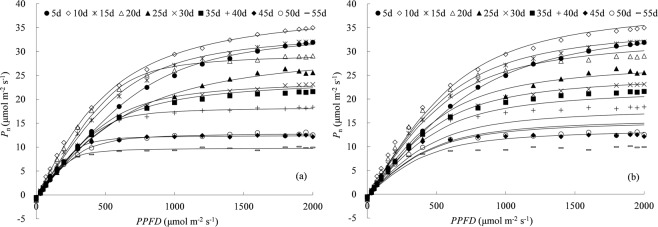
Net photosynthetic rates (*P*_n_) calculated by (**a**) the non-rectangular hyperbola (NRH) and (**b**) the general NRH equation for validation data. Lines represent the modelled *P*_n_, symbols are the observed *P*_n_ (“*n* d” in legend is the nominal leaf age for each leaf age group with ±2 days span).

**Figure 7:**
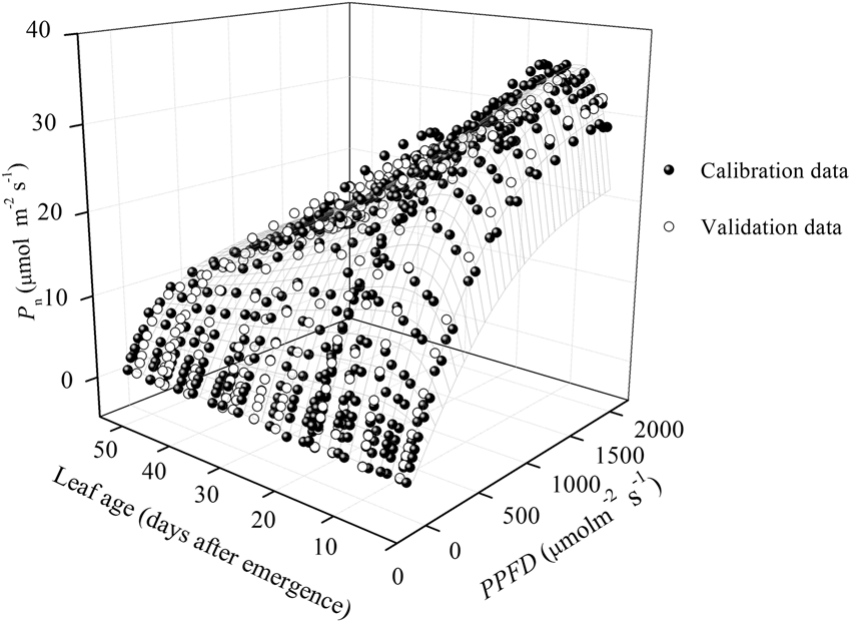
Net photosynthetic rates (*P*_n_) estimated by the general non-rectangular hyperbola (NRH) equation for rice leaves at various ages under different photosynthetic photon flux density (*PPFD*) conditions.

The other 12 PLR curves were calculated for validating the general NRH equation (Fig. 6 and Table 1). The average RMSE and AE were 0.992 and 0.918 μmol m^−2^ s^−1^ for validation of the general NRH equation, were similar to those for calibration data. The general NRH equation was capable enough of accounting the effect of leaf age on leaf photosynthesis trait, and could provide an easy way for simulating the PLR for all leaves at various ages with one set of parameters (as listed in Fig. 7). That offers a novel tool to understand variation of rice leaf photosynthetic traits along with leaf aging. Connecting the general NRH model with canopy light distribution^40^, will offer a mechanism-based method to upscale leaf photosynthesis to canopy scale. The general NRH model also provides a tool for simulating leaf photosynthesis continuously along with leaf aging by integrating it with leaf development model.

## Conclusions

Leaf age accounted for significant variation in response of net photosynthesis rates (*P*_n_) to light intensity. The parameters of light-saturated net photosynthetic rate (*P*_nmax_), quantum yield of assimilation (*φ*) and dark respiration rate (*R*_d_) in the non-rectangular hyperbola (NRH) equation were highly correlated with leaf age, whereas convexity of curve (*k*) was not. The parameters of *P*_nmax_, *φ* and *R*_d_ initially increased rapidly to a maximum around 10 days after leaf emergence, and then declined linearly with leaf age. The general NRH, which incorporated the quantitative correlations of leaf age with the parameters of *P*_nmax_, *φ* and *R*_d_ into the NRH equation, could provide a mechanistic method for simulating the photosynthetic light response curve for all leaves at various ages with one set of parameters, which will be useful for upscaling leaf photosynthesis model to canopy scale mechanically or simulating leaf photosynthesis continuously along with leaf aging.

## Methods

### Data collection

Three latest emerged leaves of rice (variety of Japonica Rice NJ46) were tagged at two-day intervals for different plants in jointing and booting stage for leaf age measurement, and leaf age was termed as the days after emergence. Thirty-seven PLR curves from 37 individual plants were obtained from 1st August to 15th September by a photosynthesis system (LI-6800; LI-COR, Lincoln, NE, USA) equipped with a red/blue LED light source (LI6800-02B) at 19 *PPFD* levels (namely 2000, 1950, 1900, 1800, 1600, 1400, 1200, 1000, 800, 600, 400, 300, 200, 150, 100, 70, 50, 30 and 0 μmol m^−2^ s^−1^), at leaf temperature of 30 °C, relative humidity of 70%, and CO_2_ concentration of 400 μmol mol^−1^. Simultaneously, leaf SPAD values were measured using the SPAD-502 (Konica Minolta, Japan).

### Non-rectangular hyperbola and its general equation

The NRH equation was fitted using a least square regression for every specific PLR curve^41^

4 $${P}_{n}=\frac{\phi I+{P}_{nmax}-{[{(\phi I+{P}_{nmax})}^{2}-4k\phi I{P}_{nmax}]}^{1/2}}{2k}-{R}_{d}$$

where *P*_nmax_ is light-saturated net photosynthetic rate; *I* is photosynthetic photon flux density; *φ* is quantum yield of assimilation, which defines the initial slope for the photosynthesis-incident light curve; *k* is the convexity of the curve; *R*_d_ is dark respiration rate. The parameters *φ* and *R*_d_ were calculated using linear regression analysis (*P*_n_ to *PPFD* < 200 μmol m^−2^ s^−1^), then *P*_nmax_ and *k* were derived empirically by fitting Eq. (4) to light response data (*P*_n_ to *PPFD* of 0–2000 μmol m^−2^ s^−1^)^42^.

Eq. (4) was first fitted separately for 37 independent PLR curves, resulting in 37 sets of coefficients of the NRH equation. The curves and coefficients were evaluated at various leaf age ranges (five-day interval). Subsequently the correlation of the parameters in the PLR equation with respect to leaf age was constructed using the 25 PLR curves (calibration data) (see Eqs. (1–3)), and were incorporated into the NRH equation to build a general NRH equation. Furthermore, the general NRH equation was validated by the other 12 independent PLR curves (validation data), which were selected randomly with a wide coverage of leaf age out of the 37 curves.

### Statistical analysis

The one-way ANOVA with the least significant difference test was used to assess the differences in photosynthetic parameters with a significance level (*p*) of 0.05. Furthermore, the performance of the NRH and general NRH equations were evaluated by the average absolute error (AE), root mean squared error (RMSE), coefficient of determination (R^2^) and Nash-Sutcliffe coefficient (NS) (Eqs (5–8)).

5 $${\rm{AE}}=\frac{1}{n}\sum _{i={\rm{1}}}^{n}|{P}_{\mathrm{ncal},i}-{P}_{\mathrm{nobs},i}|$$

6 $${\rm{RMSE}}=\sqrt{\frac{1}{n}\sum _{i={\rm{1}}}^{n}{({P}_{\mathrm{ncal},i}-{P}_{\mathrm{nobs},i})}^{2}}$$

7 $${R}^{2}={\rm{1}}-\frac{{\sum }_{{\rm{i}}={\rm{1}}}^{n}({P}_{\mathrm{ncal},i}-{P}_{\mathrm{nobs},i})({P}_{\mathrm{ncal},i}-\overline{{P}_{{\rm{ncal}}}})}{\sqrt{{\sum }_{{\rm{i}}={\rm{1}}}^{n}{({P}_{\mathrm{nobs},i}-\overline{{P}_{{\rm{nobs}}}})}^{2}}{\sum }_{{\rm{i}}={\rm{1}}}^{n}{({P}_{\mathrm{ncal},i}-\overline{{P}_{{\rm{ncal}}}})}^{2}}$$

8 $$\,{\rm{NS}}={\rm{1}}-\frac{{\sum }_{{\rm{i}}={\rm{1}}}^{n}{({P}_{\mathrm{ncal},i}-{P}_{\mathrm{nobs},i})}^{2}}{{\sum }_{{\rm{i}}={\rm{1}}}^{n}{({P}_{\mathrm{nobs},i}-\overline{{P}_{{\rm{nobs}}}})}^{2}}$$

where *P*_ncal,*i*_ and $\overline{{P}_{{\rm{ncal}}}}$ are the *P*_n_ calculated by the NRH or general NRH equation for leaves at *i* days old and the corresponding average value, *P*_nobs,*i*_ and $\overline{{P}_{{\rm{nobs}}}}$ are the observed *P*_n_ for leaves at *i* days old and the corresponding average value, *n* is the total number of *P*_n_ data.

## Supplementary information

Dataset 1

## Data Availability

All data generated during and analyzed during this study are included in this published article (and its Supplementary Information Files).
